# *Thetha Nami ngithethe nawe* (Let’s Talk): a stepped-wedge cluster randomised trial of social mobilisation by peer navigators into community-based sexual health and HIV care, including pre-exposure prophylaxis (PrEP), to reduce sexually transmissible HIV amongst young people in rural KwaZulu-Natal, South Africa

**DOI:** 10.1186/s12889-023-16262-x

**Published:** 2023-08-15

**Authors:** Jacob Busang, Thembelihle Zuma, Carina Herbst, Nonhlanhla Okesola, Natsayi Chimbindi, Jaco Dreyer, Nelisiwe Mtshali, Theresa Smit, Samkelisiwe Ngubane, Siphesihle Hlongwane, Dumsani Gumede, Ashley Jalazi, Simphiweyenkosi Mdluli, Kristien Bird, Sithembile Msane, Priscilla Danisa, Willem Hanekom, Limakatso Lebina, Ngundu Behuhuma, Cheryl Hendrickson, Jacqui Miot, Janet Seeley, Guy Harling, Jana Jarolimova, Lorraine Sherr, Andrew Copas, Kathy Baisley, Maryam Shahmanesh

**Affiliations:** 1 Africa Health Research Institute, Mtubatuba, KwaZulu-Natal South Africa; 2 Institute for Global Health, University College London, London, UK; 3 University of KwaZulu-Natal, Durban, South Africa; 4 University of the Witwatersrand, Johannesburg, South Africa; 5 Health Economics and Epidemiology Research Office, Wits Health Consortium, Johannesburg, South Africa; 6 Department of Medical Microbiology, Academic Medical Center, University of Amsterdam, Amsterdam, Netherlands; 7 London School of Hygiene & Tropical Medicine, London, UK; 8 Harvard T.H. Chan School of Public Health, Boston, USA; 9 Massachusetts General Hospital, Boston, USA

**Keywords:** Differentiated HIV prevention, HIV pre-exposure prophylaxis, Universal test and treat, Sexual and reproductive health, Adolescents and youth, Implementation trials, Peer navigators, Community-based

## Abstract

**Background:**

Antiretroviral therapy (ART) through universal test and treat (UTT) and HIV pre-exposure prophylaxis (PrEP) substantially reduces HIV-related mortality and incidence. Effective ART based prevention has not translated into population-level impact in southern Africa due to sub-optimal coverage among youth. We aim to investigate the effectiveness, implementation and cost effectiveness of peer-led social mobilisation into decentralised integrated HIV and sexual reproductive health (SRH) services amongst adolescents and young adults in KwaZulu-Natal (KZN).

**Methods:**

We are conducting a type 1a hybrid effectiveness/implementation study, with a cluster randomized stepped-wedge trial (SWT) to assess effectiveness and a realist process evaluation to assess implementation outcomes. The SWT will be conducted in 40 clusters in rural KZN over 45 months. Clusters will be randomly allocated to receive the intervention in period 1 (early) or period 2 (delayed). 1) Intervention arm: Resident peer navigators in each cluster will approach young men and women aged 15–30 years living in their cluster to conduct health, social and educational needs assessment and tailor psychosocial support and health promotion, peer mentorship, and facilitate referrals into nurse led mobile clinics that visit each cluster regularly to deliver integrated SRH and differentiated HIV prevention (HIV testing, UTT for those positive, and PrEP for those eligible and negative). Standard of Care is UTT and PrEP delivered to 15–30 year olds from control clusters through primary health clinics. There are 3 co-primary outcomes measured amongst cross sectional surveys of 15–30 year olds: 1) effectiveness of the intervention in reducing the prevalence of sexually transmissible HIV; 2) uptake of universal risk informed HIV prevention intervention; 3) cost of transmissible HIV infection averted. We will use a realist process evaluation to interrogate the extent to which the intervention components support demand, uptake, and retention in risk-differentiated biomedical HIV prevention.

**Discussion:**

The findings of this trial will be used by policy makers to optimize delivery of universal differentiated HIV prevention, including HIV pre-exposure prophylaxis through peer-led mobilisation into community-based integrated adolescent and youth friendly HIV and sexual and reproductive health care.

**Trial registration:**

*ClinicalTrials.gov Identifier*—NCT05405582. Registered: 6th June 2022.

**Supplementary Information:**

The online version contains supplementary material available at 10.1186/s12889-023-16262-x.

## Background

Despite global concerted efforts to curb the HIV epidemic, South Africa (SA) remains at the epicentre with approximately 8.2 million people in the country living with HIV. Despite effective biomedical tools, including freely available HIV testing, universal test and treat (UTT) and HIV pre-exposure prophylaxis (PrEP) there were an estimated 230,000 new infections in South Africa in 2020, the highest number in the world, with youth aged 15–24 accounting for 32% of these [1]. This high HIV incidence occurs in the context of high sexual and reproductive health (SRH) morbidity [2, 3].

Although HIV prevalence amongst youth in SA has been declining over recent years, HIV incidence among adolescent girls and young women (AGYW), particularly amongst women aged 20–24, in rural KwaZulu-Natal (KZN) remains high [4, 5]. An ambitious scale-up of combination behavioural and structural interventions in South Africa between 2016–2018 to reduce HIV among AGYW (DREAMS study) did not accelerate the decline in HIV incidence [6, 7] and the reductions in HIV incidence were explained in part by improved male HIV outcomes, due to increased AGYW male partners’ access to HIV prevention and treatment [4, 8].

HIV prevention has advanced in recent years because of new developments in biomedical HIV prevention tools including: HIV point of care tests (POCT) and self-tests [9]; the use of daily oral tenofovir/emtricitabine and most recently long acting cabotegravir for PrEP which reduce HIV acquisition by up to 90% [10]; and HIV treatment with antiretroviral therapy (ART) that eliminates onward transmission [11, 12]. However, in HIV test-and-treat studies in rural KZN fewer than one third of young men and women aged < 30 years who were diagnosed with HIV during the study went on to access HIV care [13, 14] and thus did not benefit from the advantages to their own health and remained able to pass on the virus.

There has been a consistently high burden of poor sexual health among young people in rural KZN [2]. Our recently completed study of community-based SRH among young people aged 16–29 years found 22% of participants had a curable sexually transmitted infection (STI) at baseline, and 14% acquired a new STI during the study (personal communication).

There is growing evidence on the effectiveness of community-based HIV care. A meta-analysis found that community healthcare worker HIV care delivery significantly improved HIV viral suppression, which also reduces sexual transmission [15]. The DO ART trial in KZN in 2016–2020 showed that community-based HIV test-and-treat, in which people were tested in the community and started on ART treatment without needing to visit a clinic, was superior to facility-based HIV treatment (in which once diagnosed, people need to attend a clinic for treatment) on suppressing HIV viral load, particularly among men [16]. Similarly, the SEARCH HIV UTT trial in Kenya and Uganda showed the acceptability and feasibility of universal testing and provision of risk-informed PrEP, albeit with lower uptake among young people [17].

Community-based approaches, when integrated with wider psychosocial care, foster social networks and norms that endorse HIV care and can accelerate progress towards attaining UN sustainable development goal targets [18, 19]. This is particularly important for adolescents as shown in a peer-led service delivery intervention integrated with psychosocial support among adolescents living with HIV in Zimbabwe. This was the first study that showed significant improvements in virological suppression in adolescents living with HIV in the African region [19–21].

Evidence for peer-led HIV prevention amongst key populations, such as sex workers, men who have sex with men and people who inject drugs, is well established. A 2020 systematic review of the effectiveness of peer-led out-reach amongst key populations found a 36% reduction in HIV incidence [22]. The evidence to support peer-based biomedical HIV prevention interventions amongst young people is more limited. A systematic review identified 54 peer-based interventions of HIV prevention and found twelve studies that examined peer-based interventions with young people. These included peer education in schools or participatory learning approaches to empower young people to take greater control over their relationships. They found improvements in knowledge, self-reported sexual behaviour and condom use, however none looked at biomedical interventions or health outcomes [23].

To fill this evidence gap we conducted community-based participatory research to develop and pilot the *Thetha Nami* (Talk to Me) sexual and reproductive health (SRH) intervention*.* In brief, men and women aged 18–30 years were selected by community leaders as potential peer-navigators [24]. They were trained and took part in three participatory intervention development workshops (2016–2018). The co-created *Thetha Nami* consisted of area-based peer-navigators who provided safe spaces and community advocacy and used a structured needs-assessment tool to tailor psychosocial support, peer mentorship and referral to appropriate health and social services. We found that this community-based delivery of HIV care and prevention with peer support was acceptable and feasible [24].

We hypothesise that social mobilization will attract and engage young people into decentralised SRH services where HIV prevention is tailored to need. Decentralised differentiated biosocial HIV prevention will increase uptake of risk-informed biomedical HIV care and prevention and reduce the overall prevalence of sexually transmissible HIV amongst young people aged 15–30. We are testing our hypotheses in a cluster-randomised stepped wedge trial.

## Objectives

### Main trial objective

The overarching goal of the trial is to identify scalable and sustainable ways to stem the HIV epidemic and its negative impact on young people aged 15–30 in rural KZN, South Africa through effective implementation of biosocial HIV prevention (trial registration NCT 05405582). The trial is focused on adolescents and young people, because our studies have shown a high unmet sexual health need in this age group and low engagement with existing HIV prevention and care services [2, 13, 24–30].

### Specific trial objectives

This hybrid implementation-effectiveness trial has two main objectives: (1) to measure the effectiveness of social mobilisation combined with decentralised SRH services and tailored HIV prevention in (a) creating demand for differentiated HIV prevention and care, and (b) reducing the prevalence of transmissible HIV; (2) to understand real-world implementation of social mobilisation and decentralised SRH to deliver tailored, differentiated biosocial HIV prevention, by evaluating its acceptability, feasibility, reach, scalability and cost-effectiveness.

## Methods

### Trial design

**Fig. 1:**
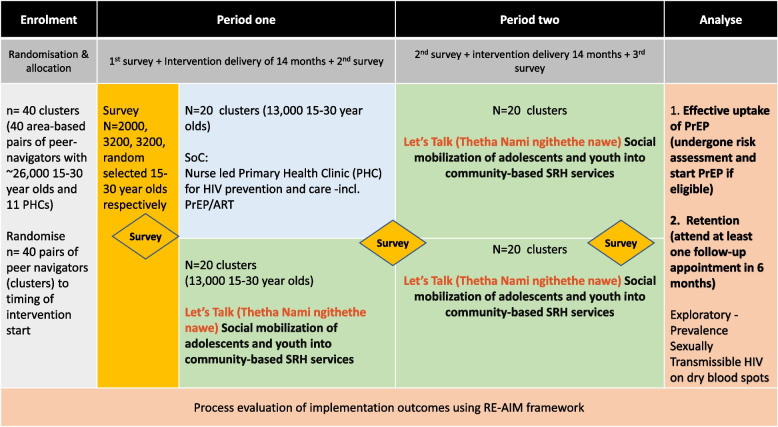
Hybrid design 1 effectiveness implementation trial design

This hybrid design trial will include a cluster randomized stepped-wedge design to assess effectiveness and a realist process evaluation to assess implementation outcomes, conducted over 45 months from 2022 to 2024 (Fig. 1). The stepped wedge trial will include three cross-sectional surveys conducted before and across two intervention periods, each of 20 months duration, and will be conducted in 40 clusters (administrative areas) in rural KZN. Clusters will be randomly allocated to receive the intervention in period 1 (early) or period 2 (delayed). A pair of resident peer navigators in each cluster will deliver the intervention to young people in the area.

**Fig. 2:**
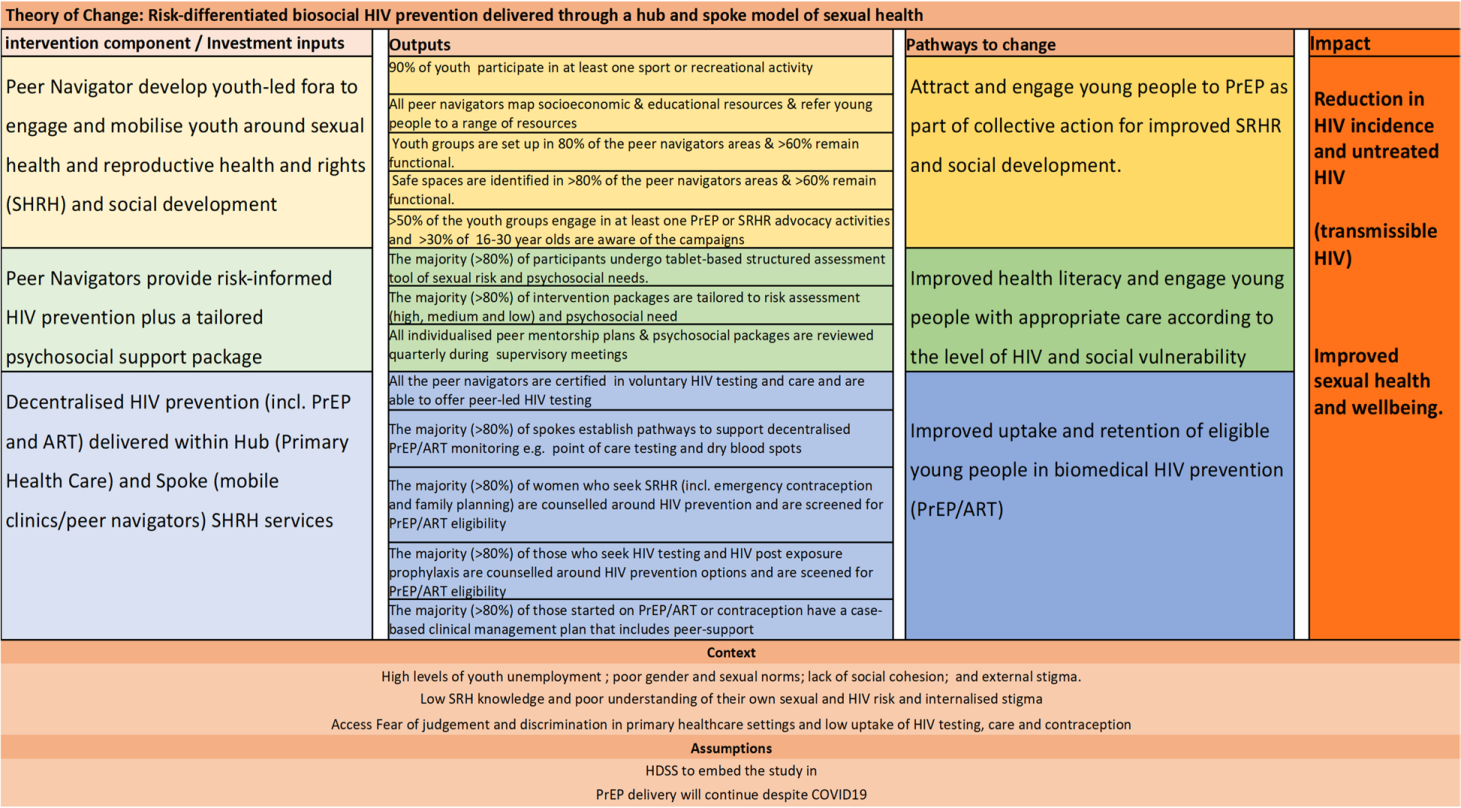
Theory of change (ToC) for Differentiated biosocial HIV prevention—Social mobilization, SRH and tailored HIV

We will use a realist process evaluation to interrogate the extent to which the intervention components deliver according to the theory of change (Fig. 2) and support demand, uptake, and retention in risk-differentiated biomedical HIV prevention, including PrEP and UTT in young people. We will use the process evaluation data to inform any modifications within the intervention arm within period one.

### Study setting

This trial is embedded in the Africa Health Research Institute’s (AHRI) multidisciplinary adolescent and youth programme in AHRI’s demographic surveillance area in uMkhanyakude district in rural KZN, SA [31]. The programme uses community-based participatory research to develop, implement and evaluate HIV prevention interventions [24–28, 30, 32, 33]. The surveillance area has a population of around 160,000, including around 26,000 young people aged 15–30 years; youth unemployment is over 85% and there is a high burden of HIV [31]. The area has 11 primary health care clinics (PHC) which have started to provide PrEP since 2021. However, uptake has been low due to poor primary care service use by young people (personal communication). All 11 clinics have implemented AHRI’s ClinicLink system, whereby clinic research assistants capture the date and reason for attendance for all consenting individuals attending the clinics. Individuals are also linked to their unique surveillance identification number at the time of the visit. By using the same unique identifier in the study, we can link young people who engage with the peer navigators or study clinics with information collected in AHRI’s tri-annual demographic surveillance. Surveillance data are further augmented through record linkage to the local health service electronic HIV care record system (TIER.Net). These clinical data collection systems mean that we can measure population-wide reach and coverage of effective HIV prevention and HIV outcomes amongst all young people aged 15–30 residing in the area.

The trial will be conducted in 40 areas (clusters) of AHRI’s surveillance area that have been purposively selected to include areas where peer navigators are working and reflect a range of rural, peri-urban and urban settings. The clusters have distinctive boundaries based on roads and rivers to minimise the risk of contamination/spill-over.

### Study population

a The population eligible to receive the intervention:
  All young people aged 15–30 years residing in 40 clusters, of whom an estimated 20% are at risk of HIV acquisition (based on our pilot work using a screening tool based on the South African national guidelines) and would benefit from PrEP, are eligible to receive the intervention.
b The eligible population for the evaluation:
  The primary outcome of the prevalence of transmissible HIV will be collected through three population-based surveys among a random sample of 16–30-year-olds who are resident in the 40 clusters: at baseline, at the end of period 1 and at the end of period 2.

### Study interventions

Clusters have been randomly allocated to receive the early or delayed rollout of the Thetha Nami intervention. All individuals allocated to the delayed intervention will receive standard of care in the first period of the trial.

#### Standard of Care (SOC)

All participants in the SOC clusters are able to access the nurse-led HIV prevention and treatment services at PHCs in the surveillance area. Standard services include HIV counselling and POCT, with immediate initiation of ART if positive, or PrEP if negative and eligible according to South African National PrEP guidelines [34] and family planning support and syndromic management for STIs as per South African National Department of Health Guidelines. Individuals who are initiated on PrEP are seen again at 1 month, and then every 3 months, for repeat HIV testing, counselling and adherence support, and prescription refills. Individuals who initiate ART are seen every 3 months for prescription refills and adherence counselling, and have viral load measurements at 6 and 12 months, then annually thereafter if suppressed.

#### Thetha Nami ngithethe nawe

The intervention is a tailored psychosocial support and social mobilisation into community-based SRH and differentiated HIV prevention, including PrEP and UTT. The intervention is provided by 90 (17 men, 73 women aged 18–30) area-based peer navigators and adolescent- and youth-friendly nurse-led SRH mobile clinics that visit fixed sites across the clusters every 2 weeks.

Peer navigators are overseen by a team of 8 peer navigator supervisors, and a committee that includes a professional nurse, a social worker, and team leads. They undergo weekly team debriefings and ongoing supervision and training facilitated by supervisors and team leads. Peer navigators deliver the following services to 15–30-year-olds in their area: providing safe spaces and community advocacy to create an enabling environment; youth groups to mobilize young people; structured psychosocial and health needs assessment using a secure electronic clinical management tool programmed into a tablet or mobile phone using REDCap software [35] to tailor support, including to young key populations (young women who sell sex, and men who have sex with men). Based on the needs assessment, peer navigators grade the needs as high (in need of immediate escalation to the committee for nurse or social worker assistance); medium (referral to clinical, educational, legal, advocacy, or social services and follow-up within a week); and low need (health promotion, provision of contact details of peer navigator and clinic hotline, and re-assess in 3 months). Peer navigators generate action plans based on the need assessment, which is electronically shared with supervisors. The action plan describes the peer-led health promotion provided and planned; referral to mobile SRH and/or other services; peer-mentorship plans with individualized (risk-informed) psychosocial support and community lay-counselling; provision of condoms, HIV self-tests and/or POCT, pregnancy tests; ART/PrEP pick-up through serostatus neutral adherence clubs with or without HIV self-test (or peer-led HIV POCT) to support decentralised PrEP. Where relevant the action plans are shared with the SRH mobile clinic via the supervisors and committee.

### SRH mobile clinic procedures

The SRH mobile clinics visit the intervention clusters every 2 weeks. The clinics deliver nurse-led HIV testing, prevention and care including adolescent- and youth-friendly, gender neutral, HIV status neutral, individualized risk assessments for HIV care and PrEP, integrated with SRH services (one stop shops). During the SRH clinic appointment, participants will receive counselling around sexual health, fertility intentions, contraception and HIV. This is part of sexual health counselling with PrEP to stay negative or ART to stay well and virally suppressed (undetectable = uninfectious). All clinic attendees are offered pregnancy testing (if female), family planning support, choice of contraception, and syndromic management for STIs, and, if male, referral to voluntary medical male circumcision. Everyone is offered HIV counselling and POCT, and immediate initiation of ART if positive. All those who are HIV negative undergo screening for PrEP eligibility according to South African National guidelines. Those who are sexually active are also offered testing for STIs, including POCT for syphilis and hepatitis B (and vaccine if negative), self-taken vaginal swabs or urine tests for gonorrhoea and chlamydia, and treatment and partner notification if positive.

If the participant agrees to immediate PrEP/ART initiation, s/he is issued with a month’s supply of tenofovir disoproxil fumarate and emtricitabine (TDF/FTC) or ART. Participants receive a phone call seven days after initiating PrEP/ART to complete a standard symptom screen for adverse effects and be referred to a fixed PHC for care if necessary. Participants have a mobile clinic appointment scheduled one month after PrEP/ART initiation. As per national guidelines, mobile clinic appointments for refills and monitoring are every 3 months thereafter; we provide community refills aiming for continuous PrEP supplies. If they agree, participants are referred back to peer navigators for adherence support and to ensure clinical follow-up. The electronic clinical management tool facilitates secure bidirectional communication of case-based action plans between the mobile clinic, the oversight committee and the peer navigator teams in the cluster.

### Primary outcomes

There are 3 co-primary outcomes: 1) effectiveness of the intervention in reducing the prevalence of sexually transmissible HIV; 2) uptake of universal risk informed HIV prevention intervention, in particular PrEP; 3) cost of transmissible HIV infection averted.

We will measure transmissible HIV as the proportion of participants aged 16–30 years providing a blood sample at endline who are living with HIV and have a detectable HIV viral load of ≥ 400 copies/mL. This composite outcome captures the effect of the intervention on both incident HIV and untreated HIV. Success of our intervention implies that there will be fewer cases of young people who acquire HIV, and those who do acquire HIV will be identified and started on treatment, both of which will reduce the number of individuals with unsuppressed (transmissible) HIV.

We will measure the uptake of universal risk informed HIV prevention intervention as the proportion of participants who are aware of their HIV status and are either on treatment if living with HIV or have taken up PrEP if HIV negative and eligible. We will measure the costs to the provider or healthcare system for each participant screened, enrolled and retained on PrEP per covered month. We will compare the costs of care in the intervention and control periods to determine the cost-effectiveness of the intervention in achieving the endpoints, i.e., the cost per transmissible HIV case averted, the cost per case linked to risk differentiated biosocial HIV prevention, cost per case linked to PrEP.

### Secondary outcomes

Secondary outcomes of the trial include: access to sexual and reproductive health services; STI prevalence; HIV incidence (measured using recency assays); proportion of men and women aged 15–30 at risk of acquiring HIV or transmitting HIV; mental health as measured by PHQ9, alcohol and drug use; and socioeconomic outcomes (educational, employment and food security). Secondary process and economic outcomes include evaluation of fidelity, acceptability, practicability, cost, uptake, and reach of the different intervention components.

### Randomisation restriction

The 40 administrative areas were randomly allocated in a 1:1 ratio to early or delayed roll-out of the intervention. Randomisation was restricted to ensure that the trial sequences were reasonably balanced with respect to several key covariates that were thought to be associated with the outcome: the population size of young people aged 15–30 years; location in the northern or southern part of the study area; proximity to a major road. The tolerance thresholds for balance were defined through an iterative process in which different thresholds were tried and the number and the validity of the acceptable allocations were examined. With unrestricted randomisation, every pair of clusters has the same probability of being allocated to the same sequence. When evaluating our restricted randomisation scheme, we aimed to obtain a sufficient number of acceptable allocations and a reasonably uniform distribution of joint allocation probabilities.

The mean population size of young people in the 40 clusters was 606. Just over half (21, 52.5%) of the clusters were in the northern surveillance area, and 9 clusters (22.5%) were along the major road. We restricted to allocation options that had absolute differences in the mean cluster size between the two sequences of ≤ 100, the proportion of northern clusters in each sequence was 40.5–64.5%, and the proportion along the main road was 12.4–32.6% (mean ± 1.5 standard deviations). Without restrictions, there were 13.8 × 10^10^ possible allocations in which 40 clusters could be randomised to early or delayed roll-out; we generated a subset of 1,000,000 of these to evaluate our restrictions. After applying the restrictions, 49.6% remained after applying these restrictions. Inspection of the validity matrix found that two larger clusters in the southern surveillance area were allocated to the same sequence 35% of the time, and two other clusters were allocated to the same sequence 54% of the time; these values were not too extreme and were considered to be acceptable.

### Randomisation and assignment of intervention

Peer navigators and other key stakeholders were invited to a public randomisation ceremony. The public ceremony ensured transparency and fairness in the randomisation and increased buy-in and engagement of the community. A random subset of 10,000 acceptable allocations was taken from those previously generated to use for the ceremony. Each allocation was given a unique running number, 1 through 10,000. Four sacks containing 10 balls each, numbered 0–9 was prepared. For each cluster, the peer navigators chose their own leader to represent their cluster. First, each leader was asked to draw a piece of paper from a box. All the papers were blank except for four. Next, the four leaders with the non-blank papers were each invited to draw a ball from one of the four sacks. The 4-digit number that was generated by this process corresponded to the allocation running number (with 0000 representing 10,000).

### Blinding

Investigators, statisticians, research assistants enrolling to the surveys, and laboratory staff will be blind to intervention assignment throughout. Participants, peer navigators and clinical teams cannot be blinded or masked.

### Criteria for discontinuing or modifying allocated interventions

In the intervention clusters, nurses and/or peer navigators will engage participants throughout the trial, and tailor their HIV prevention to their risk, including stopping and starting PrEP. Participants who experience adverse effects will be referred to a fixed PHC for care if necessary. If anyone seroconverts on PrEP (new positive ELISA test), we will support them to start ART immediately, check for HIV resistance, and switch to monitoring HIV viral loads. In the control clusters, care will be provided by the department of health nurses in PHCs according to the current South African National guidelines. All study participants will continue to receive ART/PrEP and contraception through the PHCs after the trial end.

### Strategies to improve adherence in the intervention clusters

All those who start PrEP, ART or contraception in the intervention clusters are offered peer navigator support as part of their individualized adherence plan and to support the refills and/or appointment scheduling and reminders. Neutral text message reminders are provided for participants who have access to private messaging and phone calls. A telephone hotline managed by a nurse is provided which participants can contact at any time for clinical guidance and psychosocial support.

### Outcome ascertainment

Data for outcome ascertainment are/will be collected from four sources: i) cross-sectional surveys of random samples of 15–30-year-olds who are resident in the 40 clusters; ii) programme, process, and clinical data; iii) qualitative data collected during the process evaluation; iv) records of resource utilisation (such as consumables, medicines, diagnostics) and cost data including financial reports for staff and overhead costs.

#### Cross-sectional surveys

For each cross-sectional survey, we will use the surveillance area as a sampling frame to randomly select a sample of young people stratified by sex. Our initial sample size calculations were based on sampling *n* = 3600 (90 per cluster) young people aged 15–30 years at each survey, with the expectation that 2800 would be eligible, and that 2000 (~ 50 per cluster) would be contactable and consent to participate. After the baseline survey was completed, we found that the prevalence of transmissible HIV was lower than we had anticipated. Therefore, we have decided to increase the planned sample size for the midline and endline surveys to 6000 young people (150 per cluster) per survey. This will help to maintain the power of the study despite the lower prevalence. We anticipate 4300 will be contactable and eligible and that 3200 (~ 80 per cluster) will consent to participate. The lower age limit for the survey was also increased slightly from 15 to 16 years, so that all survey participants would have been eligible for the intervention in the year before the survey.

At each survey, the study team will visit the sampled individuals at their homes to give them information about the study and invite them to participate. Those who are interested in participating will be asked to provide written informed consent if aged ≥ 18 years, or written assent and parental consent if aged < 18. Researchers will make up to four attempts at different times of the day to enrol the sampled individuals at home or a place of their choice. Based on our experience this will enable us to enrol 3200 (~ 80 per cluster) and reach our target sample size.

After informed consent, participants will be asked to provide a blood sample for HIV testing (and viral load if positive), and to complete a 20–30-min interviewer-administered questionnaire. Sensitive questions will be self-completed. Questions will include awareness of HIV status; awareness and uptake of PrEP, voluntary medical male circumcision, ART and contraception; and exposure to youth groups, peer navigators, and SRH mobile clinics. We will also collect data on sociodemographics, sexual risk (e.g., number of partners, condom use, and transactional sex), reproductive health (e.g., contraception, pregnancy, fatherhood); and mental health (PHQ9, alcohol and drug use). Participants will also be asked for their consent to link their survey data with programmatic data collected from peer navigators, SRH mobile clinics, and PHCs in the surveillance area (Table 1).

**Table 1:**
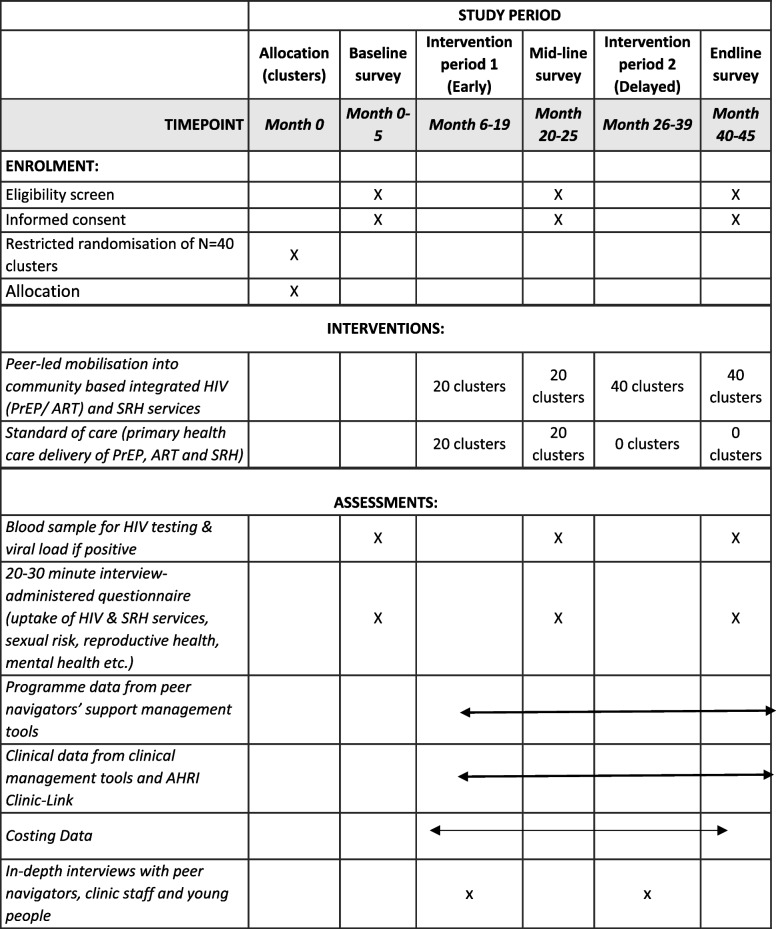
Schedule of enrolment, interventions, and assessments for Thetha Nami ngithethe nawe stepped wedge cRCT

#### Programme and process data

We will collect aggregate data on uptake and retention in the different components of the intervention in all young people aged 15–30 years who are resident in the 40 clusters (around 26,000). We will collect the programme data from the peer navigators’ participants-support management tools. Peer navigators use these tools to record psychosocial and health needs assessments, and the health promotion, service and/or referral provided. For those attending the clinics, we will collect aggregate clinical data from the clinical management tool, including HIV testing, ART uptake, PrEP eligibility screening, PrEP uptake and other services received. We will also measure retention, adherence, and reasons for stopping and/or restarting PrEP. Within the 11 PHCs in the surveillance area, we will also use the ClinicLink system to identify 15–30 year-old individuals by cluster who attend the clinics for HIV testing, PrEP or ART (Table 1).

#### Qualitative data

Qualitative data will be conducted by a team of research assistants and include in-depth interviews (IDI) with peer navigators (*n* = 10), nurses/clinical research assistants (*n* = 6) in clinics in the participating communities, and a purposive sample of young people aged 15–30 years (*n* = 50). Additionally (*n* = 1) group discussion comprising mixed age and gender community members (*n* = 4); clinical staff (*n* = 1) and (*n* = 2) peer navigators will be conducted. This will enable the researchers to understand, contextualize and explore some of the issues around the intervention (Table 1).

#### Costing data

We will work closely with the Health Economics and Epidemiology Research Office to establish the costs of implementing the intervention. We will adapt data collection tools that we have used in previous peer-led trials to measure the costs of the peer navigator intervention to collect bottom-up ingredient-based costs [29, 36]. We will complement this with a top-down costing approach using the AHRI study budgets and expenditure reports and patient cost cohorts to measure out of pocket expenditure (Table 1).

### Theory of change

It is envisaged that the intervention would reduce the burden of transmissible HIV amongst young people aged 15–30 through a reduced incidence of new cases of HIV and an increase in those with HIV who are virologically suppressed on ART. The Theory of Change (Fig. 2) shows how this would be achievable through social mobilization and SRH to create demand for HIV prevention; attracting and engaging young people to uptake HIV prevention tailored to their need; and improved uptake and retention of young people in biomedical HIV prevention (PrEP/UTT/voluntary medical male circumcision).

### Sample size

Data from our previous studies and the demographic surveillance suggested 8% of young people aged 15–30 have a transmissible HIV viral load (primary outcome 1), and around 35% are aware of their HIV status and either on PrEP or on ART with undetectable viral load (primary outcome 2). Our original design specified 2000 young people aged 15–30 (50 per cluster) interviewed in each of the three cross-sectional survey waves. Assuming an intracluster correlation coefficient (ICC) within wave of 0.1 for transmissible HIV and 0.4 for intervention uptake, and a decay (autocorrelation) between waves of 0.9 for both outcomes, this design provided 90% power to detect an increase from 35 to 47% in primary outcome 2 and 80% power to show a reduction in primary outcome 1 from 8 to 4% when comparing the intervention to standard of care [37]. The estimates of ICC are based on the coefficient of variation (k) between clusters estimated in the range 0.7–1.0 for intervention uptake seen in a trial of peer navigators in the same area [38]. However in the trial baseline survey data, approximately 6% of young people aged 15–30 have a transmissible HIV viral load; lower than we had expected (8%). To try to maintain power for the same relative reduction in primary outcome 1 we have therefore increased the size of the midline and endline surveys to 3200 (80 per cluster) young people aged 16–30. We note that a design of 3200 young people in each of the next two surveys would provide 80% power to show a reduction in primary outcome 1 from 6 to 3% when comparing the intervention to standard of care.

### Data management

Data collected by the peer navigators and clinic staff will be captured electronically on tablets using REDCap software. Automatic checks for invalid values, internal inconsistency and implausible responses will be programmed into REDCap, and additional data validation checks will be run after data collection. Data from REDCap will be uploaded to a MySQL database server within a secure server cluster at AHRI.

### Statistical analysis

To quantify the effect of the intervention on sexually transmissible HIV, we will fit a logistic regression model to data from all three survey waves to estimate the odds ratio (OR) and 95% confidence interval (CI), adjusting for design factors and survey wave, acknowledging the clustering of the data through generalized estimating equations. To increase power and also handle any chance imbalance in the distribution of HIV risk factors between exposures, we will also adjust for pre-specified predictors known to be associated with HIV transmission risk in this population at cluster and individual level.

To measure the uptake of the universal risk-informed HIV prevention intervention we will use the survey data collected at the end of intervention exposure periods, among survey participants who consent to linkage of their survey data with our programmatic and clinical data. We will calculate the proportion and 95% CI (acknowledging clustering) of participants who are aware of their HIV status and are either on treatment if living with HIV or have taken up PrEP if HIV negative and eligible. As a sensitivity analysis, we will also compare uptake of PrEP based on our programmatic data, AHRI’s Clinic Link system, and routine electronic health records (e.g. TIER.net), and compare this with what we find in the cross-sectional surveys.

To examine the cost of transmissible HIV averted, we will measure the costs in the intervention and SOC periods including HIV screening, risk assessment, PrEP initiation and adherence on PrEP. This analysis will take the cost perspective of the provider (i.e., Department of Health, implementing partners and other agencies) and include the cost of any resources used to provide services to the client irrespective of the payer. We will use different costing methods to estimate the costs of: a) services provided prior to PrEP initiation, b) PrEP provision and c) ancillary services provided alongside PrEP provision. A top down costing approach will be used to fully capture the costs which occur prior to PrEP initiation and at an above-site level, as provision of these services is not always recorded at the level of the participant. A bottom-up micro-costing of the resources used to provide PrEP and ancilliary services that are captured in the routine medical records of the participants (e.g., PrEP and other drugs dispensed, clinic visits, pharmacy, laboratory tests performed, counselling visits) will be conducted. The total and average costs of achieving each endpoint (i.e. the cost per transmissible HIV case averted, the cost per case linked to risk differentiated biosocial HIV prevention and the cost per case linked to PrEP) for each period will be calculated and an incremental cost-effectiveness ratio determined.

We will use mixed methods to explore acceptability and equity of reach; fidelity and facets of the packages that are valued and by whom (including key groups that are harder to reach e.g. mobile youth, young women who sell sex and men who have sex with men); and any unintended social harm [39–41]. This will include triangulating several sources of data to understand uptake, reach and changes along the pathway of change described in the Theory of Change (Fig. 2).

A significance level of 0.05 will be used for all inferential analyses unless otherwise stated; a correction for multiplicity will not be applied because the three outcomes reflect different trial domains (effectiveness, process and cost). A detailed statistical analysis plan will be completed before the end of data collection.

### In-depth interviews

Interviews will be based on a topic guide focusing on the experience and perceptions of the intervention. Interviews will be recorded and transcribed verbatim, with the permission of participants. Data from the IDIs will be managed for analysis using NVIVO software. The software will be used to manage categorization and coding of identified themes from the interview transcripts. Identified themes (including participants’ quotes) and interview transcripts will be reviewed and compared by the research team for inconsistencies and adequate representation of participants’ comments. A thematic analysis of all interview data will be conducted, drawing on the theory of change to categorise themes and organise the findings.

### Adverse event reporting and harms

This is an implementation study and all tests and drugs used are approved for clinical use in South Africa. All clinical care follows South African clinical guideline. The risk of harm is anticipated to be low. Adverse events (AEs) and serious adverse events (SAE) will be captured through the process evaluation, community engagement units and community advisory boards, the hotline, as well as the peer navigators and clinic staff and logged using our incident reporting form for up to up to 12 months after the start of the intervention. Reported AEs and SAEs will be monitored, categorized based on an established grading system, and followed-up accordingly by AHRI. All AEs and SAEs will be reported to the principal investigator, Trial Steering Committee, UKZN and UCL Biomedical Research Ethics Committee.

### Ethics and trial oversight

We assert that all procedures contributing to this study comply with the ethical guidelines and standards of the relevant national and institutional committees on human experimentation and with the Declaration of Helsinki. Ethical approval has been obtained from the University of KwaZulu-Natal Biomedical Research Ethics Committee (BREC/00003735/2021) and UCL Research Ethics Committee (5672/006). All staff (including peer navigators) will be provided with training on research ethics including confidentiality, voluntary participation and good clinical practice. We will ensure confidentiality at all levels of the research process, and none of our reports, presentations or articles will contain study participants identifying information. Pseudo names will be used when reporting the data particularly qualitative data. Each participant will be assigned a unique non-identifying participant identification number. Prior to their involvement in the study, participants will be provided with adequate information about the study and they will be allowed to ask questions for clarifications. Voluntary informed consent will be ensured once participants have the full understanding of the study procedures. Written informed consent will be obtained from all participants aged 18–30 years; written assent from participants aged 15–17 years, with written informed consent from their parents or guardian. All participants will have the right to withdraw from the study at any time. it will be stated clearly to participants that their refusal to participate in the study or desire to withdraw from the study will not affect them or any other health related services they are currently accessing, including peer navigator support and clinical services provided in the study mobile clinics and DoH clinics. We have established a Trial Advisory Group with clinical trials, PrEP, statistical and social science expertise to oversee the trial. This is an effectiveness trial of different models of service delivery and all tests and drugs used are approved for clinical use in South Africa. All clinical care follows South African clinical guidelines. The risk of harm is anticipated to be low and detailed in the study protocol. For the costing, additional ethical approval has been obtained from the University of Witwatersrand Human Research Ethics Committee (Medical) (HREC 220708) and Boston University IRB (H43001). All deviations from protocol, protocol modifications will be communicated to Trial Steering Committee, Biomedical Research Ethics Committees (UCL and KZN), trial registry and in the trial publications.

## Discussion

The findings of this trial will inform the scale up of peer-led social mobilization into community-based sexual and reproductive health interventions optimized to support the uptake and retention of adolescents and young adults in long term HIV treatment, HIV pre-exposure prophylaxis and other prevention, and contraception.

During the planning phase we liaised with the National and Provincial Department of Health to ensure that the interventions are designed to generate the data needed to scale-up the interventions. We established a working group of health officials that lead HIV testing, prevention and SRH in the district and a nascent technical advisory group in the KZN province. These groups helped us optimize and integrate our existing community-based PrEP delivery with the primary health care; agree the location and distribution of spokes, and the evaluation framework. The main goal of this trial is a scalable ‘decentralised hub and spoke model’ that is aligned with re-engineering of primary care, supports effective (risk-informed) use of biosocial HIV prevention (including PrEP and UTT), and provides the infrastructure to market, test, and rapidly evaluate the implementation of new products. The model is designed to improve sexual health and reduce transmissible HIV amongst young people aged 15–30 in rural KZN, South Africa. We therefore anticipate that if effective the intervention will be scaled up within the district and allow us to test the real-world implementation of newer products such as long-acting PrEP as they emerge. This will potentially harness the full potential of antiretroviral therapy to reduce HIV incidence.

## Trial registration

NCT05405582

### Supplementary Information

**Additional file 1.**

## Data Availability

Data sharing is not applicable to this article as no datasets are reported. However, following completion of the study all the datasets generated and/or analysed during the current trial will be made available in the AHRI repository at the time of publication of the primary outcome paper. Access to the datasets generated in the study will be included in all papers reporting study outcomes. Access to the full protocol and model consent forms may be available from the author.

## Abbreviations

AGYW: Adolescent Girls and Young Women
AHRI: Africa Health Research Institute
ART: Antiretroviral therapy
DSMB: Data Safety And Monitoring Board
HIV: Human Immunodeficiency Virus
IDI: In‑Depth Interview
KZN: KwaZulu-Natal
PHC: Primary Health Care Clinic
POCT: Point of Care Tests
PrEP: Pre-Exposure Prophylaxis
SA: South Africa
SAE: Serious Adverse Events
SOC: Standard of Care
SRH: Sexual Reproductive Health
STI: Sexually Transmitted Infection
SWT: Stepped-wedge trial
UCL: University College London
UKZN: University of KwaZulu‑Natal
UTT: Universal Test and Treat
